# Landscape genetics of the nonnative red fox of California

**DOI:** 10.1002/ece3.2229

**Published:** 2016-06-16

**Authors:** Benjamin N. Sacks, Jennifer L. Brazeal, Jeffrey C. Lewis

**Affiliations:** ^1^ Mammalian Ecology and Conservation Unit Veterinary Genetics Laboratory University of California, Davis One Shields Avenue/Old Davis Road Davis California 95616‐8744; ^2^ Department of Population Health and Reproduction University of California, Davis One Shields Avenue Davis California 95616; ^3^ Washington Department of Fish and Wildlife 600 Capitol Way N Olympia Washington 98501‐1091

**Keywords:** Invasive species, landscape genetics, predator control, red fox, *Vulpes fulva*, *Vulpes vulpes*

## Abstract

Invasive mammalian carnivores contribute disproportionately to declines in global biodiversity. In California, nonnative red foxes (*Vulpes vulpes*) have significantly impacted endangered ground‐nesting birds and native canids. These foxes derive primarily from captive‐reared animals associated with the fur‐farming industry. Over the past five decades, the cumulative area occupied by nonnative red fox increased to cover much of central and southern California. We used a landscape‐genetic approach involving mitochondrial DNA (mtDNA) sequences and 13 microsatellites of 402 nonnative red foxes removed in predator control programs to investigate source populations, contemporary connectivity, and metapopulation dynamics. Both markers indicated high population structuring consistent with origins from multiple introductions and low subsequent gene flow. Landscape‐genetic modeling indicated that population connectivity was especially low among coastal sampling sites surrounded by mountainous wildlands but somewhat higher through topographically flat, urban and agricultural landscapes. The genetic composition of populations tended to be stable for multiple generations, indicating a degree of demographic resilience to predator removal programs. However, in two sites where intensive predator control reduced fox abundance, we observed increases in immigration, suggesting potential for recolonization to counter eradication attempts. These findings, along with continued genetic monitoring, can help guide localized management of foxes by identifying points of introductions and routes of spread and evaluating the relative importance of reproduction and immigration in maintaining populations. More generally, the study illustrates the utility of a landscape‐genetic approach for understanding invasion dynamics and metapopulation structure of one of the world's most destructive invasive mammals, the red fox.

## Introduction

Invasive species can have detrimental effects on native communities and threatened or endangered prey populations through competition or maladaptive hybridization with closely related taxa (Rhymer and Simberloff 1996; Genovesi 2009; Doherty et al. 2015). Understanding the landscape level processes of invasions and factors maintaining invasive species is important to inform management strategies, for example, by identifying locations where control efforts are likely to be most effective (Lecis et al. 2008; Berry and Kirkwood 2010; Estoup and Guillemaud 2010; Fraser et al. 2013) and, more generally, by providing a conceptual understanding sufficient to prevent or manage future invasions.

Among invasive species, mammalian predators contribute disproportionately to declines in global biodiversity, and within this group the red fox (*Vulpes vulpes*) lists among the top‐2 species in terms of global impact (Doherty et al. 2015; the other being the house cat). Red foxes are typically monogamous, with breeding pairs maintaining exclusive territories, and young dispersing and potentially breeding in their first year (Voigt 1987). Dispersal tends to be male‐biased in terms of frequency, with females more often remaining on the natal territory as nonbreeding helpers, but distances of dispersers of both sexes can be up to 400 km or more (Allen and Sargeant 1993). In the absence of physical barriers or competitors, such as coyotes or native foxes, invading red foxes have the capacity for rapid geographic expansion (Lewis et al. 1999; Sacks et al. 2011; Abbott et al. 2014; Kasprowicz et al. 2016).

The most devastating impacts of red foxes have been associated with introductions of wild‐caught individuals to locations where the species was formerly absent (Bailey 1993; Woinarski et al. 2015). However, the rapid propagation of fur farms beginning in the early 1900s led to an explosion in the numbers of introductions (inadvertent and deliberate) of captive‐reared red foxes, particularly within the United States (Bailey 1993; Lewis et al. 1999; Long 2003; Bryce et al. 2011; Statham et al. 2012). Where they have become established, these captive‐derived (i.e., feral) foxes have impacted numerous endangered ground‐nesting bird species and threatened the genetic integrity of native red foxes through hybridization (Lewis et al. 1999; Sacks et al. 2011).

Although ultimately derived primarily from wild eastern Canadian and Alaskan ancestry, fur‐farm red foxes reflect multiple generations of selective breeding for a variety of traits such as tameness, high fecundity, and even polygyny (Dearborn 1915), which potentially increase their invasiveness and predispose them to success in human‐dominated environments. In contrast to Australian invasive red foxes, which derive from wild‐caught European individuals that spread to remote habitats throughout the continent (Statham et al. 2014), introduced farm‐reared foxes in the United States have tended to establish relatively localized populations in close proximity to humans, in urban or agricultural landscapes (Aubry 1984; Lewis 1994; Statham et al. 2012; Kasprowicz et al. 2016). In densely human‐populated regions, such as along the east and west coasts of the United States, introduced red foxes can occur over large continuous ranges interspersed with native populations, which can serve to obscure their population structure (Lewis et al. 1999; Sacks et al. 2011; Kasprowicz et al. 2016).

Development of effective management strategies is often hindered by lack of understanding about the population structure, including connectivity, specific routes of spread, relative roles of reproduction or immigration in sustaining local populations, and potential for interbreeding with native populations. Genetic tools provide a means of elucidating points of introduction, identifying hybridization with native relatives, as well as reconstructing routes of spread and assessing the relative roles of continued immigration versus reproduction in sustaining local invasive populations (Hampton et al. 2004; Lecis et al. 2008; Kidd et al. 2009; Berry and Kirkwood 2010; Estoup and Guillemaud 2010; Sacks et al. 2011; Beauclerc et al. 2013; Fraser et al. 2013).

Limited genetic studies of fox farms from throughout the world (Sacks et al. 2009; Statham et al. 2011, 2012) confirm the general understanding (e.g., Dearborn 1915) that they derive primarily from populations of eastern Canada and Alaska, with some contribution from the Washington Cascades. However, virtually nothing is known about the particular genetic composition and structure among the many 20th‐century fox farms in the United States, including the 69 farms known to have been established in California during the early 1900s (Lewis et al. 1999). It appears that most operations in the western United States were small and started from as few as one or two breeding pairs purchased from larger breeders in the East or Midwest (Westwood 1989). Thus, it seems likely that different farms contained small, potentially differentiated subsets of the available fur‐farm stock. Moreover, there are very few records of escapes or releases with which to generate specific hypotheses about the sources, origins, and routes of spread by nonnative red foxes. Therefore, most of our understanding of fur‐farm ancestry stems from genetic analyses of contemporary feral populations inferred to have been derived as escapees or releases from fur farms (e.g., Perrine et al. 2007; Sacks et al. 2010a,b; Statham et al. 2012; Kasprowicz et al. 2016). In the West, nonnative red foxes occur in low‐elevation parts of Washington, Oregon, California, Utah, Colorado, Nevada, and Idaho, although their distributions are not well documented in many of these states and often difficult to know without genetic analyses to differentiate the typically higher‐elevation native red foxes (Statham et al. 2012).

In California, nonnative red foxes were initially documented in the early to mid‐1900s in two locations 650 km apart (Southern California [SO], Sacramento area), with no evidence of expansion until the 1970s (Vail 1942; Gray 1975; Lewis et al. 1999; Sacks et al. 2011). Between the mid‐1970s and mid‐1990s (by which time, no extant fox farms occurred within California), nonnative red fox range increased to a seemingly continuous span covering an area of ~170,000 km^2^, which implied a continuous increase in area of ~20% per year (Lewis et al. 1999). While this type of exponential growth is typical of successful invading species, Lewis et al. (1999) hypothesized that it resulted from an increase in the frequency of human introductions and transplantations in the late twentieth century, rather than wholesale expansion from the one or both of the initial concentrations. In particular, the rise of the nonnative red fox in California corresponded in time to the demise of the state's fur‐farm industry, suggesting the possible role of deliberate releases from defunct fur farms (Harvey et al. 1992).

In the present study, we used 13 microsatellites and ~700 bp of mitochondrial DNA (mtDNA) of 402 nonnative red foxes collected from throughout lowland areas of California to investigate numbers of introductions, routes of spread, and contemporary connectivity among populations. The broader purpose of this study was to understand as completely as possible the “anatomy” of the red fox invasion of California both to assist in its management and to provide guidance to management of other nonnative red fox populations. Our first objective was to test hypotheses relating to the mechanics of the range increase. If the contemporary range reflected a sudden expansion from one or two locations, we expected to see a pattern of high genetic connectivity (e.g., low *F*_ST_) or isolation by distance. In contrast, if the contemporary range reflected many independent introductions of small numbers of individuals, we expected to observe substantial genetic structure with little relationship to proximity. Our second objective was to elucidate patterns of contemporary connectivity, which potentially affect the maintenance of particular populations or the entire metapopulation. To accomplish this, we applied a combination of population genetic statistics and ordination, tree‐based, and Bayesian clustering approaches to elucidating population structure free of spatially explicit models, and landscape resistance surface modeling approaches that tested explicit habitat‐based hypotheses about connectivity. We also investigated whether some populations were dependent on immigration (“sinks”) from other populations (“sources”) for persistence (Pulliam 1988).

## Materials and Methods

### Samples

**Figure 1:**
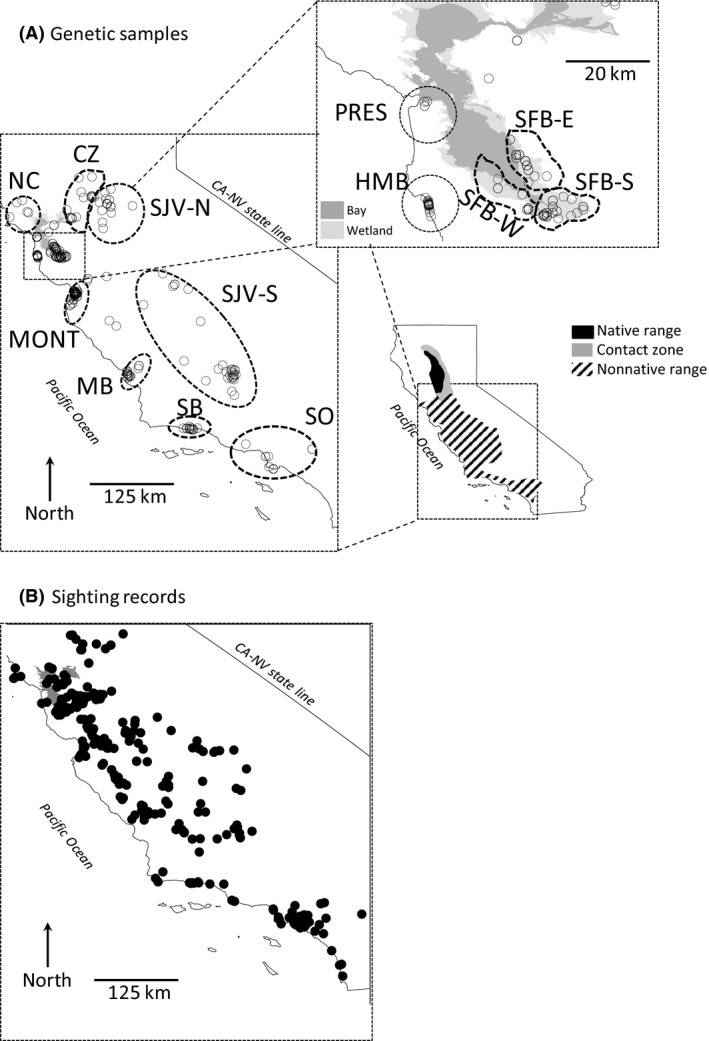
Distribution of nonnative red fox locations from central and southern California (SO), including (A) 402 genetic samples collected for this study and (B) 349 high‐reliability sighting records independently assembled by Lewis et al. (1993) and used in the present study to construct a landscape resistance model. (A) Inset of California, illustrating ranges of native, nonnative red foxes, and their contact zone (CZ); dashed polygons indicate the following sampling sites: North coastal (NC), native–nonnative contact zone (CZ), San Joaquin Valley (SJV) north (‐N) and south (‐S), Monterey (MONT), Morro Bay (MB), Santa Barbara (SB), SO, and, in the inset (upper right), Presidio of San Francisco (PRES), Half Moon Bay (HMB), and the San Francisco Bay wetlands (SFB) south (‐S), east (‐E), and west (‐W). Miscellaneous samples not associated with the 13 primary sampling sites are also shown.

We obtained most of our sample over a 15‐year period spanning 1996–2010, and small numbers of additional samples from as far back as the early 1900s, which enabled us to investigate stability of genetic patterns to more directly assess these postestablishment dynamics. We obtained samples for genetic analyses primarily from foxes removed in predator control activities aimed at protecting endangered prey species. As a consequence, most of our sampling reflects some spatial clustering in localized sampling sites (Fig. 1A). For convenience, we therefore used discrete sampling sites as a basis of several analyses, although we did not consider sites to represent biologically meaningful population units. The dispersion of samples varied among sites, in some cases reflecting somewhat arbitrary groupings (e.g., San Joaquin Valley [SJV‐S]); the grouping of such samples with a particular site was decided independently of genetic data, based solely on considerations of sample size, proximity, and commonality to a landscape (e.g., within the same valley). Consequently, it is likely that some sites contained multiple populations and some populations were spread across multiple sites. In total, we sampled 402 red foxes from 13 sites scattered across most of the known range of the nonnative red fox in California. These sites encompassed the range of nonnative red fox occurrence identified by Lewis et al. (1999), except the southernmost extent of San Diego, where foxes apparently were extirpated, and most of the Sacramento Valley, which was subsequently (to that study) found to contain the native Sacramento Valley red fox (Perrine et al. 2007; Sacks et al. 2010a,b; Fig. 1A). We included samples from the southern end of the Sacramento Valley, a known contact zone between native and nonnative red foxes (Sacks et al. 2011). After necropsy and tissue sampling, vouchers for many of these samples (*n* = 157) were accessioned in the UC Berkeley Museum of Vertebrate Zoology or other collections (*n* = 16). Data for these and all unaccessioned (i.e., all) samples were deposited in the Dryad Digital Repository (DOI No.: doi: 10.5061/dryad.hj722). For the purposes of distribution modeling, we used an independent data set of 349 red fox occurrence records that were obtained by Lewis et al. (1993) through a statewide survey of wildlife biologists and managers, including rigorous screening for reliability (Fig. 1B).

### Laboratory procedures

We conducted DNA extraction, polymerase chain reaction (PCR) amplification, sequencing, and genotyping at the Mammalian Ecology and Conservation Unit of the Veterinary Genetics Laboratory of University of California, Davis. We extracted DNA from tissue (*n* = 379) and bone (*n* = 4) specimens using the DNeasy^®^ tissue kit (Qiagen Inc., Valencia, CA), and from scats (*n* = 19) using the QIAamp^®^ Stool Kit (Qiagen, Inc.). Primers, PCR chemistry, and cycling conditions for the mtDNA D‐loop and cytochrome *b* loci were as previously reported (Perrine et al. 2007; Aubry et al. 2009; Sacks et al. 2010a,b; Statham et al. 2012) as were those for the 13 microsatellite loci. We included all microsatellite loci used by Sacks et al. (2010a,b), except for FH2001, which exhibited a null allele. All mtDNA analyses were based on a 696‐bp portion of the mitochondrial genome composed of 354 bp of the cytochrome *b* gene and 342 bp of the D‐loop. These subsets were used in previous analyses (e.g., Perrine et al. 2007; Aubry et al. 2009; Statham et al. 2012, 2014), facilitating direct comparison.

### Within‐population analyses

We estimated the mtDNA haplotype frequencies and gene diversity for each sampling site (Nei 1987). Using microsatellites, we estimated observed and expected heterozygosity, allelic richness, *F*_IS_, and tested for deviations in Hardy–Weinberg and gametic equilibrium using permutation tests in FSTAT (version 2.9.3.2; Goudet 1995), followed by sequential Bonferroni corrections (Rice 1989). We estimated the genetic effective population size based on the bias‐corrected linkage disequilibrium method (Waples 2006) implemented in LDNE (Waples and Do 2008). We assumed random mating based on evidence for a high frequency of mixed‐parent litters in other lowland foxes (Converse 2012), excluded alleles with <0.05 frequency, and used jackknife‐based confidence intervals (Waples and Do 2008). To assess signatures of demographic bottlenecks owing to founder effects, we tested microsatellites for heterozygote excess relative to expectation under mutation–drift equilibrium using program Bottleneck v 1.2.02 (Piry et al. 1999). We relied primarily on the 2‐phase mutation model assuming 70% stepwise mutations, but also report significance with respect to the infinite alleles model (IAM) and stepwise mutation model (SMM; Cornuet and Luikart 1996). We used two‐tailed Wilcoxon tests to assess statistical significance.

### Population structure

To characterize population structure, we computed a matrix of pairwise genetic distances (Nei's *D*_A_; Takezaki and Nei 1996) and used these values to generate a neighbor‐joining tree, with bootstrap values calculated from 999 resampling cycles on loci using program Populations 1.2.30 (O. Langella, 1999; http://bioinformatics.org/~tryphon/populations/). For comparison to other studies, we computed standard allele‐frequency‐based estimates of *F*_ST_ for both mtDNA haplotypes and microsatellites in Arlequin 3.1 (Excoffier et al. 2005). We estimated the ratio of male to female gene flow using the global *F*_ST_ estimates for mtDNA and microsatellites as described by Hedrick et al. (2013, eq. 7c). For use in the population genetic distance‐based analyses described below, we linearized the mtDNA estimates of *F*_ST_ as follows: *F*_ST_/(1 −* F*_ST_) (Rousette 1997). To visualize relative genetic distances among individuals, we used a principle coordinates analysis (PCoA) based on genotypic covariance, implemented in Genalex (Peakall and Smouse 2006).

### Landscape‐genetic analyses

To assess connectivity relative to the landscape, we employed empirical and model‐based approaches using population genetic distances for both microsatellites (*D*_A_) and mtDNA (*F*_ST_/[1 −* F*_ST_]). First, to visualize barriers and corridors affecting gene flow based solely on genetic distances (i.e., independently of any a priori model of landscape resistance), we used an approach similar to that by Keis et al. (2013) whereby we mapped and interpolated residuals from a regression of pairwise genetic distance on Euclidean distance (in km). This approach effectively exposed locations corresponding to greater‐than‐ or less‐than‐expected genetic distances between sampling points, while removing any potential influence of separation distance. We used the centroids of sampling sites, rather than individuals, as sample units to avoid biases associated with our spatially clustered samples. We mapped the midpoints between each pair of sites and used inverse distance‐weighted averaging among their associated residual values (i.e., observed minus expected genetic distance) to assign interpolated values to raster layer covering the study area. We examined general concordance of apparent barriers/corridors with major landscape features, rather than evaluating statistical support for any particular putative barrier or corridor. To assess whether such general correspondence could reflect chance, we conducted several permutations of midpoints to randomize them with respect to their associated genetic distances, simultaneously permuting rows and columns (Legendre 2000), and interpolated each permutation as described above (Fig. S1).

Next, we used the independent data set of 349 red fox occurrence records (Lewis et al. 1993) to derive a species distribution model, which we inverted for use as a hypothetical resistance surface to test against our genetic distance data (Fig. 1B). The occurrence records were based on telephone interviews with wildlife professionals and vetted for reliability on the basis of the interviewee's experience with the species, accuracy of the physical description, and documentation of exact date and location (Lewis et al. 1993). We developed the species distribution model using Maxent (v. 3.3; Phillips et al. 2006) to relate occurrences to the following landscape variables: Elevation, Shrubland, Forest, Woodland, Grassland, Urban‐Agriculture, and Wetland (Appendix S1). Although occurrence reports likely reflected some bias toward locations where individuals spent most of their time, the wildlife professionals interviewed worked in a variety of habitats, many of which were remote. Therefore, we doubt that such biases would have been severe enough to substantially misrepresent the underlying habitat associations of the foxes, particularly given the relatively broad extent and coarse grain of our analysis. Because our purpose was to obtain a model that improved our understanding of gene flow on the landscape, rather than to understand habitat associations mechanistically, the ultimate value our model (i.e., resistance surface) depended on its ability to predict connectivity.

We evaluated the fit of this resistance surface based on correlation to genetic distances, which was an independent data source from that used to construct it. We used program Circuitscape to produce matrices of pairwise landscape resistance based on our landscape resistance model (McRae et al. 2008). We employed simple and partial Mantel tests to assess correlations between resistance matrices, Euclidian distance matrices, and genetic distances, specifically to assess whether the resistance model explained genetic distance significantly better than did Euclidian (geographic) distance. We conducted the Mantel tests in program Passage 2 because it uses an unbiased permutation method to assess significance (Legendre 2000; Rosenberg and Anderson 2011). Euclidean and resistance distances were log‐transformed prior to analysis (Rousette 1997).

### Metapopulation dynamics

To investigate the directionality of gene flow between sampling sites, we used an assignment approach similar to that by Berry and Kirkwood (2010). Although the Bayesian method implemented in BIMr is, in principle, a more comprehensive approach to inferring directional gene flow patterns among sites within a metapopulation (Faubet and Gaggiotti 2008), our preliminary attempts to use this method produced inconsistent results, most likely because our sample size for most sites was below the recommended minimum (*n *≥* *50 per site). We therefore used program Structure (v. 2.0) to cluster samples on the basis of genotype frequencies and then examined the spatial distribution of cluster assignments (Pritchard et al. 2000). In a structured population, each cluster would be expected to correspond to a particular sampling site and migrants could be identified as individuals assigning to a cluster other than that in which they were sampled. A sink population could then be characterized as one with many individuals assigning to one or more external sites, whereas a source population would contain individuals primarily assigned to the home population. In the case of extinction–recolonization dynamics, we would expect to see the cluster assignments change over time within a sampling site.

All Structure runs were conducted assuming admixture with correlated allele frequencies (Pritchard et al. 2000; Falush et al. 2003). After 10 replicate runs of 20,000 Markov Chain Monte Carlo (MCMC) cycles (first 10,000 discarded as burn‐in) at each number of clusters (*K*), we performed a final run at each *K* consisting of 550,000 cycles (the first 50,000 discarded). We tested increasing values of *K* until the ln *P*(*D*) either decreased or became notably more variable among replicate runs for 2 consecutive values of *K*. It is common to analyze patterns in “log probabilities of the data” associated with choices of *K* to infer the “correct” or “best” number of clusters describing structure of a population (e.g., Evanno et al. 2005). However, doing so to the exclusion of alternative choices of *K* can be misleading, particularly if populations are structured hierarchically (or are structured in other ways that deviate from a simple island model). Therefore, we developed an approach here that integrates multiple levels of *K* into “cluster profiles” characteristic of each sample, and then uses the most common cluster profiles among samples to assess hierarchical structure (Appendix S2, Figs. S2, S3). To optimize our ability to infer migration among sites, we chose the highest *K* for which assignments nested within those at lower levels of *K*. To assess potential influences of uneven sample size on results, we ran analyses with a smaller random subsample from the more heavily sampled sites and found little difference from the complete data set (Appendix S3, Figs. S5–S8).

## Results

### Mitochondrial data set

We obtained mitochondrial sequences and/or microsatellite genotypes from 402 red foxes from 13 predefined sampling locations, including two 1920s foxes from the native–nonnative contact zone. We obtained 392 full cytochrome *b* and D‐loop mitochondrial sequences (Table 1). All except one haplotype, A‐273, had been previously described. This haplotype (A‐273) differed from haplotype A‐63 by 1 substitution in the D‐loop fragment, which was deposited in GenBank (Accession No. KU244024).

**Table 1:** Gene diversity and distribution of 10 mitochondrial haplotypes discovered in 392 red foxes from 13 samples and miscellaneous sites in California. Sampling sites include the following abbreviations: Half Moon Bay (HMB), Santa Barbara (SB), and San Joaquin Valley (SJV). The particular location of 10 samples from somewhere in the San Francisco Bay wetlands (SFB) was unknown (unk)

Samples	*n*	Gene diversity	D‐19	O‐26	N‐7	G‐38	A‐273	E‐9	F‐9	F‐12	F‐14	K‐36
SFB South	44	0.428	–	1	9	1	–	–	–	32	1	–
SFB East	54	0.205	–	–	48	2	–	–	1	3	–	–
SFB West	17	0.208	–	–	2		–	–	–	15	–	–
SFB‐unk	10	–	–	–	2	4	–	–	–	4	–	–
Half Moon Bay	24	0.517	–	7	2	15	–	–	–	–	–	–
Monterey	115	0.235	–	1	4	100	–	–	–	10	–	–
Morro Bay	19	0.100	–	–	1	18	–	–	–	–	–	–
Santa Barbara	13	0.000	–	–	–	–	–	–	–	–	–	13
SJV North	33	0.691	–	–	5	14	–	–	–	10	4	–
SJV South	28	0.513	–	–	7	3	–	18	–	–	–	–
Southern CA	9	0.593	–	–	–	–	2	–	5	2	–	–
Contact zone	9	0.593	5	–	1	3	–	–	–	–	2	–
North coastal	5	–	–	–	–	5	–	–	–	–	–	–
Presidio	4	–	–	3	–	1	–	–	–	–	–	–
Miscellaneous	6	–	–		–	4	–	–	1	1	–	–
Total	392	0.726	5	12	81	170	2	18	7	77	7	13

**Figure 2:**
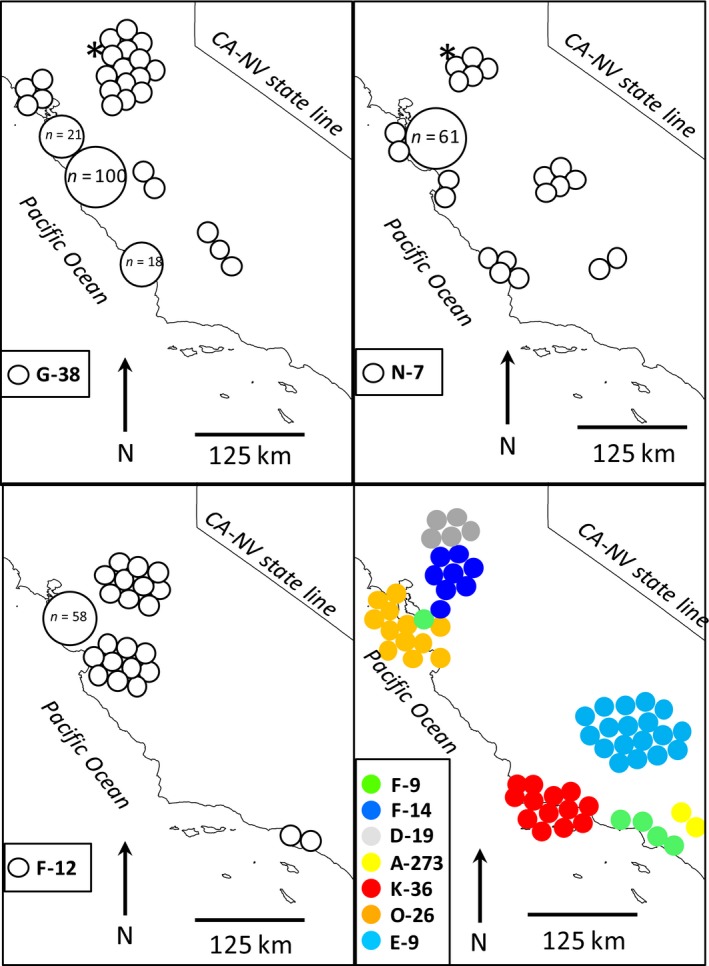
Distribution of 10 mitochondrial haplotypes from 392 red foxes sampled from central and southern California. Top and left panels each depict a single relatively widespread haplotype, whereas the bottom right panel shows the distribution of seven localized haplotypes. All haplotypes are nonnative except for D‐19, which is endemic to the native Sacramento Valley red fox and found only in the contact zone between these and nonnative red foxes. Asterisks indicate the locations of two red foxes sampled from near Davis, California, in the 1920s.

The gene diversity was high for the total sample (0.73) relative to gene diversities within sampling sites ($\overset{¯}{X}$ = 0.37, standard deviation [SD] = 0.23; Table 1). This pattern corresponded to a global estimate of *F*_ST_ = 0.49 (i.e., 1 − 0.37/0.73), and all but three haplotypes were restricted to ≤3 sampling sites, indicating considerable population structure (Fig. 2). Pairwise *F*_ST_ estimates averaged 0.54 (range: 0–1; Table S1). The two most widespread haplotypes were G‐38 and N‐7, which also were found in two specimens (UC Davis, Museum of Wildlife and Fish Biology, Catalog Nos. 10z, 17z) collected in the 1920s in southern Yolo County near an active fur farm (Lewis et al. 1999).

### Microsatellite data set

We obtained genotypes from 381 of the 402 red foxes in 13 sampling sites, including 10 sites with 9–115 foxes each (Table 2). In total, we observed 106 alleles across 13 loci for which we genotyped 10–13 loci each (average No. loci = 12.8 loci). No single locus showed significant deviation from Hardy–Weinberg or gametic equilibrium in any sampling site. However, in the entire sample combined, all loci deviated significantly from Hardy–Weinberg equilibrium and 27 of 78 locus pairs deviated from gametic equilibrium, indicating considerable population structure in the total data set. The estimate of global *F*_ST_ (theta) was 0.086 (95% CI: 0.074–0.098). Pairwise *F*_ST_ estimates averaged 0.10 (range: 0.01–0.18; Table S1). Using the formula of Hedrick et al. (2013) with mtDNA and nuclear global *F*_ST_ estimates indicated a ratio of male to female gene flow of 4.2.

**Table 2:** Population genetic statistics based on 13 microsatellite loci for 349 nonnative red fox from 10 sampling sites in California, including expected (assuming Hardy–Weinberg equilibrium) heterozygosity (*H*_e_), observed heterozygosity (*H*_o_), allelic richness (AR), inbreeding coefficient (*F*_IS_), genetic effective population size (*N*_e_), and significance (**, ***) of the Wilcoxon test for heterozygote excess indicative of a population bottleneck

Sampling site	*n*	*H*_o_ (SE)	*H*_e_ (SE)	AR (SE)	*F*_IS_	*N*_e_ (95% CI)	Bottleneck
South SF Bay	44	0.51 (0.02)	0.60 (0.05)	2.3 (0.1)	0.15*	21.7 (14.8–33.3)	**
East SF Bay	54	0.51 (0.02)	0.61 (0.05)	2.4 (0.1)	0.17*	18.4 (12.7–27.2)	**
West SF Bay	17	0.52 (0.03)	0.55 (0.05)	2.2 (0.1)	0.06	14.8 (7.7–37.0)	***
HMB	24	0.64 (0.03)	0.66 (0.05)	2.5 (0.2)	0.04	13.9 (8.6–24.2)	***
Monterey	115	0.64 (0.01)	0.69 (0.03)	2.6 (0.1)	0.08*	44.6 (28.9–73.0)	***
Morro Bay	18	0.57 (0.03)	0.60 (0.04)	2.3 (0.1)	0.05	2 (1.6–2.5)	***
SB	11	0.60 (0.04)	0.65 (0.04)	2.4 (0.1)	0.07	6.3 (2.9–13.1)	***
SJV North	29	0.67 (0.02)	0.68 (0.04)	2.6 (0.1)	0.02	12.6 (8.9–18.3)	***
SJV South	28	0.58 (0.03)	0.66 (0.05)	2.5 (0.1)	0.12*	31 (18.2–69.9)	**
Southern CA	9	0.56 (0.05)	0.71 (0.04)	2.6 (0.1)	0.22*	23.8 (10.9–260.4)	**

HMB, Half Moon Bay; SB, Santa Barbara; SJV, San Joaquin Valley; SE, standard error; IAM, infinite alleles model; SF, San Francisco; SMM, stepwise mutation model.

*P < 0.05; **P < 0.01 for IAM and TPM; not significant (*P *>* *0.05) for SMM; ***P < 0.01 for IAM and TPM; *P *<* *0.03 for SMM.

**Figure 3:**
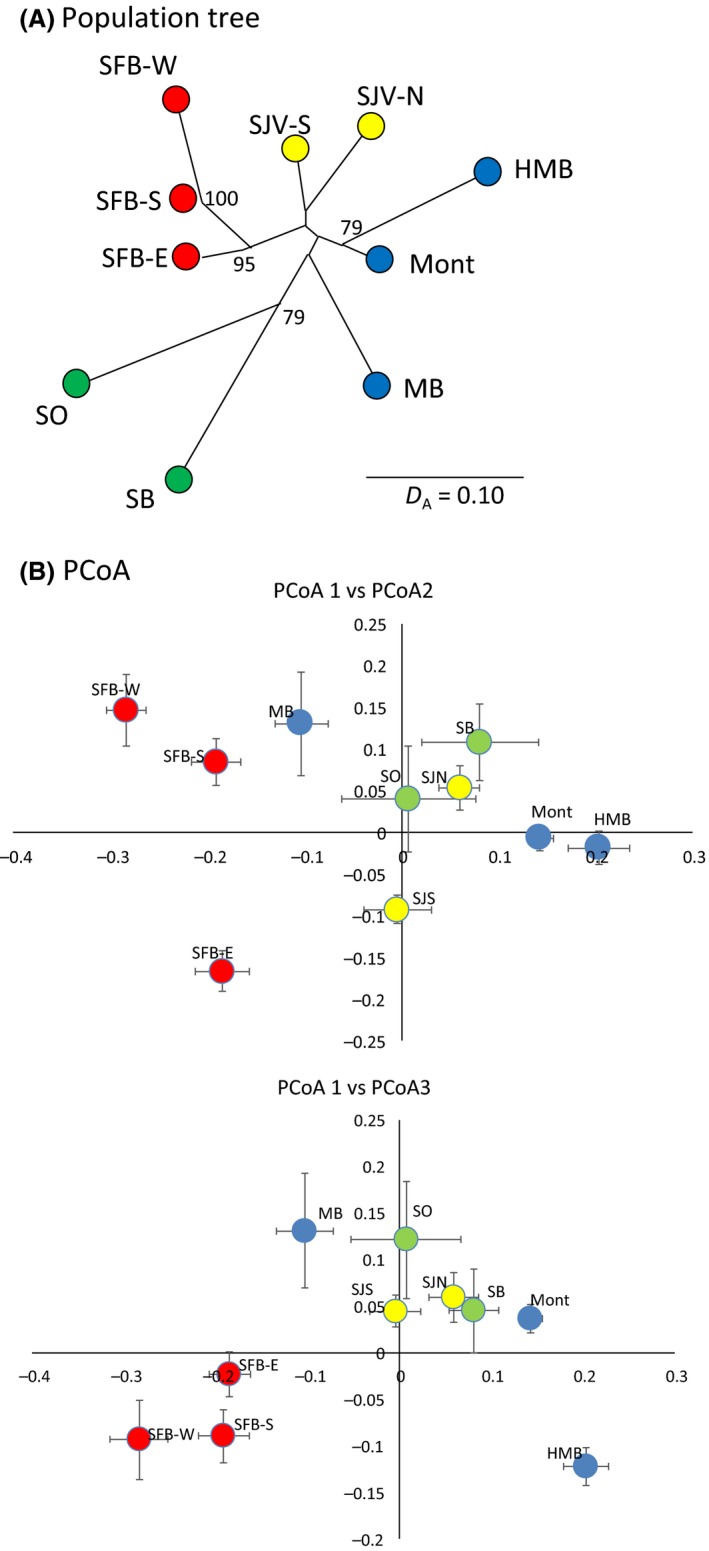
Genetic distances among 10 nonnative red fox sampling sites estimated from 13 microsatellite loci (*n *=* *349 foxes), including (A) an unrooted neighbor‐joining tree based on Nei's *D*_A_ with bootstrap support >65% indicated, and (B) principle coordinates (PCoA), including centroids and standard errors along two axes. Sampling sites were San Joaquin Valley (SJV) north (‐N) and south (‐S), Monterey (Mont), Morro Bay (MB), Santa Barbara (SB), Southern California (SO), Half Moon Bay (HMB), and the San Francisco Bay wetlands (SFB) south (‐S), east (‐E), and west (‐W). Samples were color‐coded for convenience to distinguish sites of the San Francisco Bay area (red), central coast (blue), inland (yellow), and south coast (green).

Observed heterozygosity was lowest in the three San Francisco (SF) Bay Area sampling sites (0.51–0.52) and highest in the northern SJV (0.67), followed closely by Monterey and Half Moon Bay (0.64 each) sites (Table 2). Expected heterozygosity (ranging 0.55–0.71) and allelic richness (ranging 2.2–2.6) were less variable, with most estimates falling within 2 standard errors of others. Five sites showed no statistically significant heterozygote deficiency, whereas five sites exhibited *F*_IS_ values significantly greater than zero (ranging 0.08–0.22), suggesting the presence of admixture. Genetic effective population size estimates ranged from 2 (95% CI: 1.6–2.5) in Morro Bay to 44.6 (28.9–73.0) in Monterey. All populations showed signatures of demographic bottlenecks, consistent with founder effects (Table 2). The neighbor‐joining tree based on Nei's *D*_A_ indicated 3 sets of sites that clustered with moderate to high bootstrap support: (1) the SF Bay sampling sites; (2) Half Moon Bay and Monterey; and (3) Santa Barbara (SB) and SO (Fig. 3A). Additionally, within the SF Bay sampling sites, the West SF Bay and South SF Bay clustered together relative to the East SF Bay, consistent with a clockwise stepping‐stone pattern of founding around the Bay. Except for the SF Bay populations, the positioning of populations relative to one another were consistent with their spatial arrangement on the landscape and, although not well supported by bootstrapping, both SJV sampling sites clustered together in the final tree. The PCoA similarly grouped the three SF Bay sites together as relatively distinct from the other sites (Fig. 3B).

### Connectivity across the landscape

**Figure 4:**
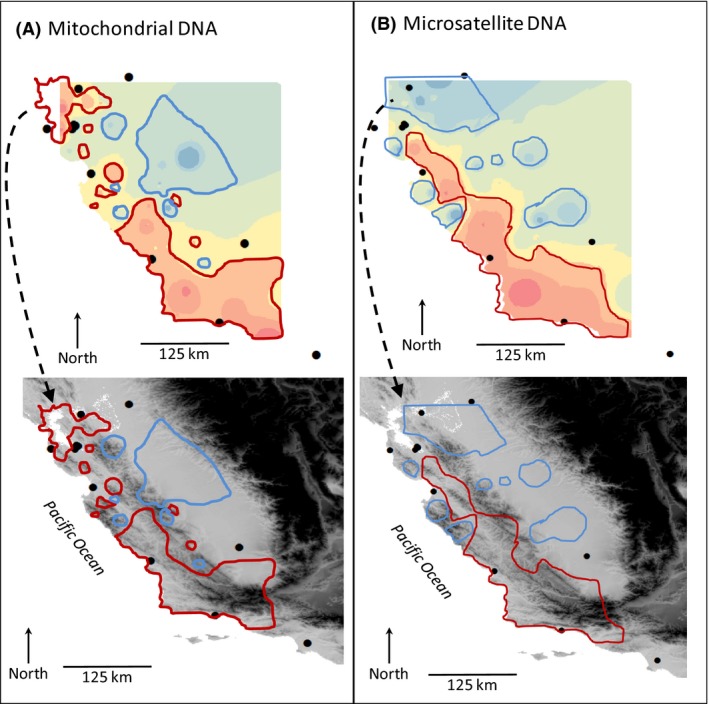
Empirical resistance surfaces inferred from inverse distance‐weighted averaging among pairwise midpoints and their associated Euclidian distance‐adjusted genetic distances (A) *F*_ST_/(1 − *F*_ST_) for mtDNA and (B) *D*_A_ for microsatellites. Interpolated surface with illustrative resistance contours highlighted by red lines and connectivity contours highlighted by blue lines are shown on top, with the same contours overlaid on elevation below. Sampling site centroids are shown as filled circles.

Interpolation of surfaces from spatially explicit genetic distances produced highly concordant results between mitochondrial (Fig. 4A–C) and microsatellite (Fig. 4D–F) markers. Superimposing these model‐free surfaces over topographic relief showed a correspondence between low gene flow and mountainous terrain (coastal mountains) and between high gene flow and flat terrain (SJV; Fig. 4). Generally, the valley habitats promoting high gene flow also corresponded to urban and agricultural habitats. Permutations indicated low cumulative areas of genetic barrier and no meaningful or consistent geographic patterns for either marker, effectively ruling out the possibility that the observed patterns and their agreement between markers were artifactual (Fig. S1).

**Figure 5:**
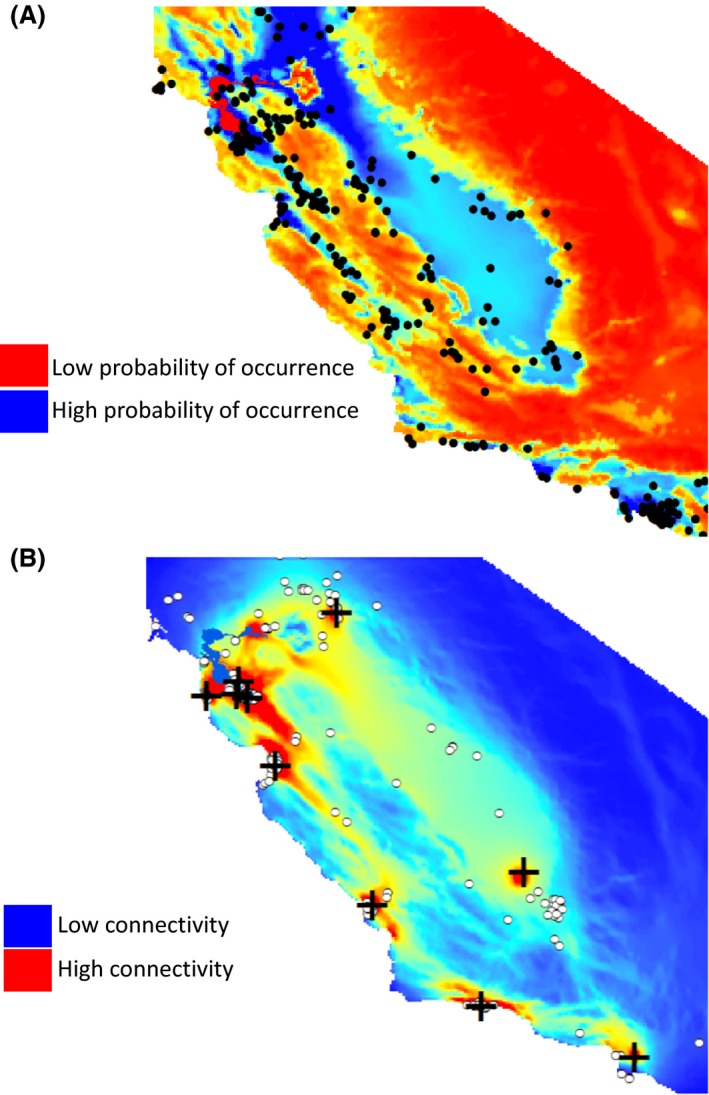
Landscape models, including (A) a Maxent species distribution model based on locations of 349 sighting reports (filled circles) from Lewis et al. (1993) and (B) connectivity (“current”) map estimated using Circuitscape, along with 402 red fox genetic sample locations (open circles) and 10 corresponding centroids (+) used to test the resistance model.

The Maxent model based on the independently collected sighting records predicted the highest probability of occurrence in the low‐elevation, flat urban and agricultural habitats of the Central Valley and smaller coastal valleys, with low occurrence in the coastal and interior mountains (Fig. 5A). Although our use of incidental visual observations likely entailed some bias toward habitats where interviewees spent the most time, the high consistency of the habitat associations, including virtual absence of sightings in the higher mountainous habitats, supports the model, qualitatively. More importantly, the purpose of this model was to serve as a hypothesis for the rules governing landscape connectivity, which we tested with independent data.

Specifically, we used the inverse of this model as a resistance surface with which to project a hypothetical connectivity (or, in circuit‐theory parlance, “current”) map (Fig. 5B). Confronting the model with genetic data indicated a substantial improvement over the use of Euclidian distance as a predictor of genetic distance. Specifically, simple Mantel tests were significant for correlations between microsatellite genetic distance (*D*_A_) and both landscape resistance (*r *=* *0.73, *P *<* *0.0001) and Euclidian distance (*r *=* *0.54, *P *<* *0.001), but the partial Mantel test was significant only for landscape resistance with Euclidian distance held constant (*r *=* *0.65, *P *<* *0.001), but not for Euclidian distance with landscape resistance held constant (*r *=* *−0.35, *P *=* *0.98). The mtDNA‐based correlations were nonsignificant for landscape resistance and Euclidian distance in both simple and partial Mantel tests.

### Metapopulation dynamics

To infer source–sink relationships between sampling sites and whether populations were extirpated and then replaced by colonists from other populations, we first conducted an admixture analysis to elucidate meaningful population units from sampling sites. In this analysis, the greatest increase in posterior probability per increase in *K* corresponded to *K *=* *2 (Figs. S2–S4, Appendix S2), which grouped all of the 3 SF Bay sites into one cluster, consistent with the previous analyses showing the SF Bay area to be distinct. However, the posterior probability continued to increase approximately linearly with increasing levels of *K*, indicating additional structure nested within each of these *K *=* *2 primary clusters (Fig. S2). Clusters nested hierarchically up to *K *=* *8, except for the Morro Bay sampling site, which did not consistently associate with any other particular sampling site (Figs. S3, S4, Appendix S2). This exception could have stemmed from the very small genetic effective size (*N*_e_ = 2) of the Morro Bay population, which would be expected to result in rapid differentiation from the founding and other populations.

**Figure 6:**
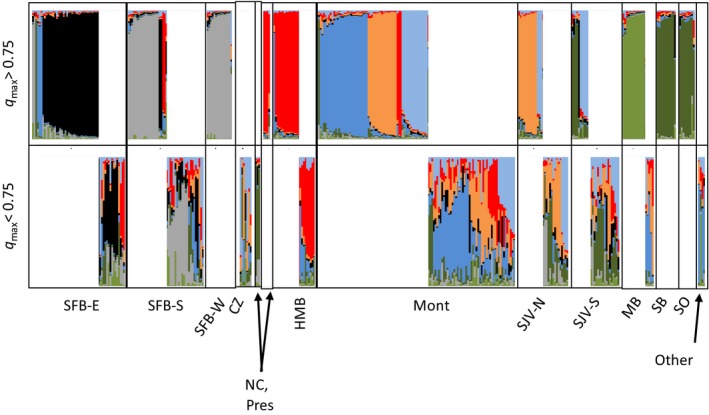
Genotypic assignments with bar graphs indicating the ancestry fraction (*q*) apportioned to individual genotypes of nonnative red foxes from California, showing 221 individuals with high assignment to a single cluster (*q*_max_ > 75%, top) and 160 admixed individuals (*q*_max_ < 75%, bottom) in 1 of *K *=* *8 clusters according to admixture analysis in program Structure. Clusters indicated by light and dark shades of the same colors indicate those clustered together at *K *=* *6 and 7 (green) or *K *=* *6 (blue).

At *K *=* *8, most sampling sites were characterized by at least one cluster representative of the home population (Fig. 6). In some cases, these home clusters also predominated in adjacent sampling sites, suggesting they reflected the same population: (1) the South and West SF Bay, (2) Presidio and Half Moon Bay, and (3) SB and SO. In the first two cases, mtDNA haplotype frequencies supported subsuming of the sites in a single population, but SB and SO did not share any mtDNA haplotypes, suggesting these were distinct populations, at least maternally, despite clustering together with microsatellites (Table 1).

Next, to determine source–sink and extirpation–recolonization dynamics, we investigated symmetry in cluster sharing both irrespective of time (a static view) and, for samples spanning sufficient timeframes (Table 3), with respect to changes over time (a dynamic view). We first examined the genotypes that assigned primarily to a single cluster (*q* > 0.75) and were therefore most likely to expose first‐generation migrants (Fig. 6). Monterey Bay, which was initially established by 1980 (Lewis et al. 1993), appeared to be a sink population, receiving immigrants from several external populations. Except for the one cluster that was nearly unique to Monterey Bay (dark blue, Fig. 6), genotypes sampled in this population primarily assigned to SJV (light blue, orange) or Half Moon Bay/Presidio (red). Additionally, many individuals in Monterey assigned only partially (<75%) to an immigrant cluster, suggesting they were progeny of first‐generation migrants. Conversely, however, there was little evidence of first‐ or second‐generation migration from Monterey into any of the surrounding populations. The exception was three individuals sampled in the East SF Bay that assigned as first‐generation migrants from Monterey, each of which was sampled after 2003 (see below). Examination of the clusters in time indicated that the proportion of immigrants in the Monterey sample approximately doubled during 2002–2007 compared to 1997–2000 (Fig. S9). Interestingly, however, the same mtDNA haplotype (G‐38) remained the most prevalent throughout these periods, suggesting male gene flow was primarily responsible for the shift in cluster assignment over time (Fig. S10).

**Table 3:** Temporal distribution of 384 nonnative red fox samples from California (an additional 18 were undated)

Sample site	Time period							
	Pre‐1980	1981–1985	1986–1990	1991–1995	1996–2000	2001–2005	2006–2010	>2010
East San Francisco (SF) Bay	–	–	–	–	45	3	3	–
South SF Bay	–	–	–	1	32	8	3	–
West SF Bay	–	–	–	–	16	–	1	–
Contact zone	2	–	–	–	–	–	6	4
North Coast	–	1	–	–	–	–	4	–
Presidio	–	–	–	–	–	4	–	–
Half Moon Bay	–	–	–	–	–	12	12	–
Monterey	–	1	–	–	43	43	21	–
San Joaquin Valley (SJV) North	–	–	–	–	2	8	23	1
SJV South	–	–	–	–	3	18	6	1
Morro Bay	–	–	–	–	–	7	11	–
Santa Barbara	–	–	1	3	1	1	7	–
Southern California	–	–	3	–	2	4	–	–
Other	–	–	–	1	10	2	4	–

The SJV South site shared a cluster with SB and SO (dark green, Fig. 6), suggesting northward migration into the SJV, but there was no evidence of reciprocal gene flow from SJV (e.g., light blue cluster) in the two more southerly sampling sites. The East and South SF Bays apparently exchanged small numbers of dispersers with one another (black, gray). In addition to the three individuals in the East SF Bay assigning to Monterey Bay mentioned above, we sampled two individuals from the South SF Bay that assigned to the Presidio/Half Moon Bay cluster. Additionally, one individual in the East SF Bay was assigned to the SO/SB cluster, and also carried the F‐9 mtDNA haplotype, otherwise found only in Los Angeles (Fig. 2); given the distance and landscape resistance, human‐assisted translocation seems the most likely explanation. Importantly, although most individuals from the SF Bay included in our study were sampled prior to 2003 (Table 3), all six of the individuals assigning as immigrants to the SF Bay area were sampled after 2003 (and these composed 75% of the eight individuals sampled from the SF Bay after 2003). Thus, the SF Bay populations could have been essentially extirpated and recolonized. Otherwise, for most populations sampled over spans of 15–20 years, we observed little change in cluster assignment or haplotype frequency over time, suggesting that most populations sustained themselves reproductively and that extirpation–recolonization dynamics were the exception (Appendix S4, Figs. S9, S10).

## Discussion

Understanding how invasive predators spread, establish, and maintain their populations is fundamental to managing their impacts. The feasibility of eradication or local control of invasive populations depends on their abundance, connectivity, and population growth rates (Bomford and O'Brien 1995; Adams et al. 2014). In the present study, we used landscape‐genetic approaches to reconstruct a nonnative red fox invasion and to characterize the postestablishment metapopulation structure and dynamics, which also provided insights about the relative importance of demographic resilience versus immigration in enabling populations to withstand predator control measures. Below, we review our key findings and then revisit previous localized control efforts in the context of our findings.

### Invasion dynamics

Prior to our study, the mapping of occurrence records of nonnative red foxes showed them to have increased from two isolated locations 650 km apart in the 1970s to many locations in‐between, which, when viewed coarsely, appeared to reflect a large continuous population (Lewis et al. 1999). The rate of increase also was consistent with exponential growth and expansion or, alternatively, a long lag period followed by a relatively sudden “explosion.” In contrast to predictions of the exponential expansion model, we found high localization of most mitochondrial haplotypes, which suggested multiple, independent sites of introduction, rather than spread from a single (or two) point source(s). Thus, our findings support the suggestion by Lewis et al. (1999) that the range increase fed off continuous introductions rather than proceeding solely of its own demographic volition. More concretely, by identifying a minimum number of populations on the basis of private haplotypes and then using shared haplotypes and dates of fox arrival to various locations to infer directionality of spread, we propose that at least eight founding populations led to the current distribution: SJV‐N, Mont, San Francisco Bay (SFB)‐E, SFB‐S/W, Presidio, SJV‐S, SB, and SO. Given our small sample size from SO and apparent haplotype heterogeneity, it seems likely that multiple introductions occurred there as well.

Nevertheless, the question remains as to the explanation for the relatively sudden appearance of red foxes over such a widespread region. We hypothesize that this population explosion was ignited by releases of foxes from defunct fur farms in multiple locations, a practice that apparently began in the 1960s in response to the economic downturn associated with the industry (Harvey et al. 1992). In contrast to episodic translocations by rehabilitators or other miscellaneous parties, which may have been common throughout the past century (Lewis et al. 1999), the large‐scale release of multiple individuals from the same captive population would have significantly increased the probability of successful reproduction and establishment. Once initial populations became established, such as in the Monterey Bay area, SB, and in the SF Bay area, smaller, miscellaneous translocations could then coalesce with, or recruit from, these initial sources to seed new populations. The possibility of dispersers pairing with other dispersers of the opposite sex in locations previously unoccupied by foxes would become increasingly likely, potentially fueling new populations. For example, Morro Bay, which was the newest population we sampled (i.e., the only one not known a decade earlier; Lewis et al. 1993), had an estimated *N*_e_ of 2 (95% CI 1.6–2.5), suggesting it could have been founded by a single pair or possibly a single pregnant female.

### Contemporary connectivity

Our findings further suggested that, although populations clearly exhibited some level of connectivity, the magnitude of gene flow was relatively low. First, the low diversity of mitochondrial haplotypes we observed within sites suggests that founders were few and slow to spread from their sources. Nuclear gene flow, although higher than mitochondrial, also was relatively low among sampling locations. For example, the average microsatellite‐based *F*_ST_ measured among sampling sites in the present study was >0.10, with several pairs of adjacent sites exhibiting estimates ≫0.10; in contrast, between the northern and southern ends of the Sacramento Valley native red fox population (spanning ~200 km), *F*_ST_ averaged <0.05 (Sacks et al. 2010b).

The presence of even limited gene flow (primarily nuclear) among populations enabled us to investigate aspects of the landscape preventing or promoting connectivity as well as the directionality of gene flow between neighboring sites. The observed correspondence between model‐free genetic distance surfaces and topographic features for both mtDNA and microsatellite markers suggested a highly fragmented metapopulation. This pattern was corroborated by modeling the association of occurrence records – a completely independent data set – with landscape variables, which provided a more highly resolved map of the predicted distribution (i.e., occurrence habitat). The confrontation of this model with genetic data, which confirmed its utility for also representing dispersal habitat, indicated that human‐dominated valleys were the primary dispersal corridors and less human‐dense mountains, the primary barriers. The affinity of nonnative red foxes for human‐dominated valleys may stem partly from their feral nature and consequent ability to thrive in disturbed habitat (e.g., Kaprowicz et al. 2016). However, aversion to mountain foothills also has been observed in the native Sacramento Valley red fox (Sacks et al. 2011) and therefore likely reflects non‐human‐related factors. In particular, competition from native canids (coyotes, *Canis latrans*; gray foxes, *Urocyon cinereoargenteus*) could be considerably greater in the foothills, as has been suggested for other lowland fox species (Nelson et al. 2007).

The resistance surface model also was insufficient to fully explain the observed population structure, in particular, the hierarchical relationship indicated by the population tree, Structure, and PCoAs. Sampling locations in the SF Bay area clustered more closely with one another than they did with other nearby sampling locations that were separated by habitat otherwise predicted by the landscape resistance model to facilitate gene flow (i.e., low‐elevation, human‐dominated landscape). The observed genetic distinctiveness of the SF Bay area populations could relate to the original sources founding them, to the lack of gene flow after their establishment, or to both. One particular mechanism potentially constraining gene flow was natal habitat‐biased dispersal, that is, the tendency to disperse into familiar habitat (Sacks et al. 2004; Stamps and Swaisgood 2007). Specifically, it is possible that individuals born within the salt marsh wetland habitat of the SF Bay dispersed solely within the wetland landscape, rather than emigrating to the highly distinct dryland habitats of the adjacent populations, and vice versa.

### Metapopulation dynamics and relation to predator management

Our genetic findings with respect to symmetry of gene flow or replacement are best interpreted in the context of control programs. The clearest example involved the relatively isolated populations of the SF Bay, where 80–100 foxes per year were removed as part of a predator control program beginning in 1992 (Harding et al. 2001). In the present study, we genotyped most of the foxes removed in 1996 and 1997, enabling us to characterize the genetic composition of the population at that time. Foxes sampled through 2002 continued to be dominated by those assigning to that population, suggesting that despite the large numbers of foxes removed each year, the population was able to maintain itself through reproduction rather than immigration. On the other hand, the small numbers of individuals we sampled between 2004 and 2007 were dominated by immigrants from coastal areas and the SJV, suggesting that control efforts eventually succeeded in reducing, and possibly eradicating, the original population. After our SF Bay area samples were collected for the present study (10/24/1995–3/31/2007), numbers of red foxes removed from this area continued to decline, suggesting that the intensity of control eventually was sufficient to overcome immigration (Foerster et al. 2011).

Red foxes also were removed from the Monterey area to protect snowy plovers, beginning in the early 1990s and continuing throughout our study, including 118 individuals removed from 1993 through 1999 (Neuman et al. 2004). Our sample included those foxes removed beginning in 1997. Our sample from the reporting period of Neuman et al. (2004) was primarily composed of two clusters and after that point continued to show these clusters, plus a significant component assigning to another cluster, which was otherwise shared with the northern SJV and could have represented unsampled locations between these sites. Although our data were insufficient to estimate the relative influence of immigration and mortality, field data on snowy plover nesting success in response to the removal efforts suggested that predator control had a net effect of reducing predator abundance (Neuman et al. 2004). Thus, it appears that in both of these populations where predator removal efforts were most intensive, immigration was increasingly frequent, compensating to some extent for the increased mortality, but depression of the populations also was possible. In the future, the use of genetic data to assess origins of individuals removed could be helpful in strategically removing individuals from contributing populations or in low‐elevation choke points along dispersal corridors (e.g., as per our model).

### Sex‐biased dispersal in relation to spread versus contemporary connectivity

We estimated a fourfold difference in gene flow attributable to dispersing males relative to females, suggesting that males were the primary agents of gene flow, at least once populations became established. However, the correspondence between genetic distance and landscape features in both types of markers (e.g., Fig. 4) suggested that the relatively weak mitochondrial footprint on connectivity was nevertheless real. This faint signature could have resulted primarily from the preestablishment period when newly released females, like males, would have had to roam to find locations to settle and breed. Previous studies also have found males to be the primary dispersers, and that frequency of dispersal is especially high among males when population density is lowest (Allen and Sargeant 1993; Lewis 1994; Gosselink et al. 2010).

### Management implications

The approach and resources developed in this study can aid local wildlife managers in planning future control activities. The landscape resistance model can be used to identify locations where predator control efforts can be most efficacious in reducing immigration or preventing recolonization. Similar approaches have been used with invasive American mink (*Neovison vison*) in Scotland (Fraser et al. 2013) and feral pigs in Australia (Hampton et al. 2004). The genetic data can also be used in the context of the landscape resistance model to identify potential eradication units (Adams et al. 2014). However, it would be important to obtain additional samples from intervening locations where foxes are likely to occur but where no control efforts are being employed (and, hence, we had no samples in the present study). In particular, two major valleys (Salinas, southern Santa Clara) east of Monterey Bay were known to contain nonnative red foxes (Lewis et al. 1993, 1999) but were not sampled in the present study. It seems likely that these populations contributed migrants to the Monterey Bay and possibly the SF Bay populations. Additionally, our findings that at least two populations changed in genetic composition over time indicate the need for continued genetic monitoring of foxes from the same sites to identify changes. For example, a relatively consistent genetic signature, such as we observed in the south and west SF Bay during 1995–2002, suggests that population persistence was most attributable to demographic compensation, whereas major changes in genetic composition, such as occurred in the same population after 2003, suggested that immigration eventually became the primary engine of persistence. Differentiating between these demographic processes is critical for identifying where future control efforts are best directed.

## Data Accessibility

DNA sequence: GenBank accession KU244024. Sampling locations, dates, types, mtDNA haplotypes, and microsatellite genotypes: Dryad doi: 10.5061/dryad.hj722.

## Conflict of Interest

None declared.

## Supporting information


**Appendix S1.** Landscape resistance surface.
**Appendix S2.** An approach to uncovering hierarchical population structure.
**Appendix S3.** Influence of uneven sample size.
**Appendix S4.** Cluster assignments over time.
**Table S1**. Pairwise *F*_ST_ estimates among 10 nonnative red fox sampling sites in California.
**Figure S1.** Inverse distance‐weighted (IDW) interpolations of residual genetic distances.
**Figure S2.** Relationship between average log probability of the data and the number of clusters (*K*) in 10 replicate runs of Structure in all foxes.
**Figure S3.** Cumulative frequency of genotypes relative to numbers of cluster profiles in all foxes.
**Figure S4.** Hierarchy diagram of cluster profiles in all foxes.
**Figure S5.** Relationship between average log probability of the data and the number of clusters (*K*) in 10 replicate runs of Structure in a subset of foxes.
**Figure S6.** Cumulative frequency of genotypes relative to numbers of cluster profiles in a subset of foxes.
**Figure S7.** Hierarchy diagram of cluster profiles in a subset of foxes.
**Figure S8.** Bar graph indicating the ancestry fraction (*q*) apportioned to individual genotypes in a subset of foxes.
**Figure S9.** Bar graphs indicating ancestry fraction (*q*) in red foxes in different sampling periods.
**Figure S10.** Bar graphs indicating mtDNA haplotypes in red foxes in different sampling periods.
