# Supplementary material for: Landscape genetics of the nonnative red fox of California
Source: Ecol Evol. 2016 Jun 16;6(14):4775–91. doi: 10.1002/ece3.2229 (PMC4979706; doi:10.1002/ece3.2229)
Supplement: Supplementary file 1 — Appendix S1. Landscape resistance surface. Appendix S2. An approach to uncovering hierarchical population structure. Appendix S3. Influence of uneven sample size. Appendix S4. Cluster assignments over time. Table S1. Pairwise F ST estimates among 10 nonnative red fox sampling sites in California. Figure S1. Inverse distance‐weighted (IDW) interpolations of residual genetic distances. Figure S2. Relationship between average log probability of the data and the number of clusters (K) in 10 replicate runs of Structure in all foxes. Figure S3. Cumulative frequency of genotypes relative to numbers of cluster profiles in all foxes. Figure S4. Hierarchy diagram of cluster profiles in all foxes. Figure S5. Relationship between average log probability of the data and the number of clusters (K) in 10 replicate runs of Structure in a subset of foxes. Figure S6. Cumulative frequency of genotypes relative to numbers of cluster profiles in a subset of foxes. Figure S7. Hierarchy diagram of cluster profiles in a subset of foxes. Figure S8. Bar graph indicating the ancestry fraction (q) apportioned to individual genotypes in a subset of foxes. Figure S9. Bar graphs indicating ancestry fraction (q) in red foxes in different sampling periods. Figure S10. Bar graphs indicating mtDNA haplotypes in red foxes in different sampling periods. [file ECE3-6-4775-s001.docx]

**Supporting Information**

**Appendix S1 – Landscape resistance surface**

We developed the species distribution model using Maxent (v. 3.3; Phillips et al. 2006) to relate occurrence reports to the following landscape variables: Elevation, Shrubland, Forest, Woodland, Grassland, Urban-Agriculture, and Wetland. These 6 vegetation types combined 73 more highly resolved vegetation types defined previously (Parker and Matyas 1979), but updated from more recent (1990s) 1:250,000-scale source maps with minimum mapping unit, 1.6 km^2^ (<http://portal.gis.ca.gov/geoportal/>, accessed 8/18/2014). Except for Elevation, which was expressed in terms of standard deviations from the mean, habitat variables (i.e., vegetation types) were expressed as dummy variables.

We used ArcGIS (v10.0; Environmental Systems Research Institute, Redlands, CA) to project locations of the 349 red-fox reports and 10,000 randomly generated points and coded them with respect to vegetation types (0,1) and Elevation (standard deviations from the mean). For vegetation types, this involved joining point features with attributes of a polygon in a vector-based layer (shapefile). For elevation, this involved using the Extract Values to Points tool in Spatial Analyst Tools. We exported the input (n = 349) and background (n = 10,000) coded points for use in Maxent modeling software in sites-with-data (SWD) format. We followed recommendations of Phillips and Dudík (2008) for model specification (e.g., we used “hinge” features, and ß = 0.5). The projections were output in the “logistic” form, which constrained predictions between 0 (low probability of occurrence) and 1 (high probability of occurrence). To create shapefiles of the model, we used ordinary kriging to interpolate projections of 91,252 random points into a raster (output cell size = 465 m), which was then clipped using Spatial Analyst Tools to remove the region corresponding to the Pacific Ocean. This was then exported as an ascii raster.

To convert this ascii raster (coded in terms of conductance) into resistance layers for landscape-genetic modeling in Circuitscape, we used the reciprocal values of 3 transformations: value, value^0.5^, and value^0.25^ in an attempt to tune the model. We assessed the fit of each of all three tuning variants of the resistance surfaces and found very little difference. We therefore presented results of analyses based only the initial model (i.e., with no tuning).

References:

Parker I, Matyas WJ (1979) CALVEG: A classification of Californian vegetation. U.S. Dep. Agric., For. Serv., Reg. Ecol. Group. San Francisco.

Phillips SJ, Anderson RP, Schapire RE (2006) Maximum entropy modeling of species geographic distributions. *Ecological Modelling*, 190, 231-259.

**Appendix S2 – An approach to uncovering hierarchical population structure**

We employed a heuristic approach using Structure to assess multiple levels of *K* based on consistency across choices of *K* without the need to pick a single “correct” level. Our premise was that some genotypes will cluster with others consistently across levels of *K*, whereas others may maintain less consistent affinities across levels of *K*. The most consistently clustering genotypes also are expected to be those least admixed and most reflective of true clusters (regardless of the value of *K*). Therefore, as long as a reasonable proportion of the genotypes maintains consistent relationships, i.e., group with at least a subset of those with which they grouped at *K*-1, these genotype groupings should be most informative about overall population structure.

To obtain a general sense of the likelihood profile, we ran structure 10 times each at *K* = 1–10, calculated average and standard errors for the log probability of the data [LnP(X)] for each value of *K*, and chose a subset of potentially informative *K* values on the basis of two criteria: (1) the value was higher than lower values of *K* and (2) probability values were consistent (small standard errors) relative to other *K* values. This procedure resulted in the greatest increase in LnP(X) between *K* = 1 and *K* = 2, suggesting that *K* = 2 corresponded to a primary division in the data, but LnP(X) continued to increase essentially linearly to *K* = 8, above which LnP(X) became more variable (Fig. S2). Therefore, we chose *K* = 2–8 as levels of analysis to explore. We reran Structure at each of these *K* values using 50,000 burn-in cycles and 500,000 post-burn-in cycles.

Next, we composed for each genotype a “cluster profile” reflecting cluster assignments at each successive level of *K*. For example, the cluster profile, 2-3-4-2-1-1-8, corresponded to an individual assigned to cluster 2 at *K* = 2, cluster 3 at *K* =3, cluster 4 at *K* = 4, cluster 2 at *K* = 5, cluster 1 at *K* = 6, cluster 1 at *K* = 7, and cluster 8 at *K* = 8. Using cluster profiles for genotypes with q > 75% on average (across levels of *K*), we then tallied the frequency of cluster profiles and noted that the top 11 most-frequent cluster profiles described 90% of all genotypes (Fig. S3).

We diagrammed these most-frequent 11 cluster profiles to determine which levels of *K* were consistently nested within lower levels of *K* (Fig S4). Cluster assignments were perfectly nested (i.e., hierarchical) at 6 $\leq$ *K* $\leq$ 8 (Figure 6, main text). A cluster profile that failed to nest at *K* < 6 corresponded to genotypes from Morro Bay.

**Appendix S3 – Influence of uneven sample size**

Because Structure assignments can be influenced by uneven samples sizes, we conducted a second analysis in which we randomly subsampled 20 of the 54 East SF Bay samples, 20 of the 61 South and West SF Bay samples, and 20 of the 115 Monterey Bay samples. The plot of the log probability of the data was similar to the one for the entire data set (Fig. S5) as was the cumulative frequency of cluster profiles, except that 6 cluster profiles was sufficient to represent >80% of the “pure” (Q > 75%) genotypes (Fig. S6). We then diagrammed the top 6 cluster profiles as above to determine which levels of *K* were consistently nested within lower levels of *K* (Fig S6). As above, cluster assignments were perfectly nested (i.e., hierarchical) at 6 $\leq$ *K* $\leq$ 8. Barring the less-frequent cluster profiles associated with *K* = 8 assignments to cluster 5, the hierarchical structure also extended to 2 < *K* < 6 (Figure S7). The only notable differences in this analysis (Figs. S7, S8) from the analysis that utilized all 381 genotypes (Fig. S4) were that (1) the cluster 5 (orange) at *K* = 8 had inconsistent affinities at lower levels of *K* with clusters 1, 3, 6, and 8, (2) the structure was less well defined for the San Francisco Bay sites and in the Monterey Bay, where the sample was reduced, and (3) Morro Bay remained distinct from Santa Barbara and Southern California at *K* = 6, 7, and 8.

**Appendix S4 – Cluster assignments over time**

After apportioning admixture of the 381 red foxes to *K* = 8 clusters as described above (e.g., Figs S4), we divided 7 of the site-specific samples into time periods to examine temporal dynamics, such as replacement of a recipient population by founders from another population (Fig. S9). Other sampling sites were sampled for sufficiently short periods that we could not meaningfully subdivide them temporally, but their assignments are shown in Figure 6 (main text) and the dates of sampling can be found in Table 3 in the main text.

The geographic distribution of cluster assignments during the first period of sampling, approximately 1990–2000, was similar to that represented in the total sample, providing little evidence of wholesale population replacement, at least during the well-sampled periods. Some minor changes apparent in latter samples, such as the disappearance of the light-blue cluster (c8) from Monterey and the SJV North sites and an increase in the cluster diversity in the SJV South site, apparently reflect minor and gradual, local changes. Additionally, the increased diversity in the SJV South site coincided with a broader sampling of the SJV; most of the dark green (c3) and light blue (c8) samples in all periods derive from in and around Bakersfield, whereas the differing cluster assignments associated with one of the latter periods correspond to samples from further north.

However, the SF Bay, which we sampled primarily before 2004, could have experienced extirpation and re-establishment after this time. In contrast to the relatively strong self-assignments of foxes in this region prior to 2004, the assignments of 6 of 8 individuals sampled from the SF Bay sites during 2004–2007 were assigned to external clusters. Indeed, one of these individuals was assigned to the dark green cluster (c3) and also carried an F-9 mitochondrial haplotype, found otherwise only in Southern California, suggesting the possibility of human-assisted translocation.

**Supporting Tables**

Table S1. Pairwise *F*_ST_ estimates among 10 nonnative red fox sampling sites in California based on 13 microsatellite loci (above diagonal) or mitochondrial haplotype frequencies (below diagonal). Sample sizes (mtDNA\microsatellites) are indicated in the diagonal.

|  | South SF Bay | East SF Bay | West SF Bay | HMB^a^ | Monterey | Morro Bay | SB^a^ | SJV^a^ North | SJV^a^ South | Southern CA |
| --- | --- | --- | --- | --- | --- | --- | --- | --- | --- | --- |
| South SF Bay | 44\44 | 0.05 | 0.01 | 0.13 | 0.08 | 0.11 | 0.14 | 0.08 | 0.08 | 0.09 |
| East SF Bay | 0.59 | 54\54 | 0.09 | 0.14 | 0.08 | 0.12 | 0.16 | 0.09 | 0.06 | 0.1 |
| West SF Bay | 0 | 0.75 | 17\17 | 0.18 | 0.12 | 0.13 | 0.17 | 0.12 | 0.11 | 0.12 |
| HMB^a^ | 0.5 | 0.63 | 0.6 | 24\24 | 0.06 | 0.14 | 0.13 | 0.11 | 0.09 | 0.1 |
| Monterey | 0.65 | 0.75 | 0.74 | 0.18 | 115\115 | 0.1 | 0.1 | 0.04 | 0.04 | 0.06 |
| Morro Bay | 0.71 | 0.84 | 0.89 | 0.25 | 0.03 | 19\18 | 0.14 | 0.11 | 0.1 | 0.1 |
| SB^a^ | 0.69 | 0.84 | 0.88 | 0.67 | 0.79 | 1 | 13\11 | 0.1 | 0.1 | 0.09 |
| SJV^a^ North | 0.23 | 0.49 | 0.31 | 0.13 | 0.26 | 0.31 | 0.55 | 33\29 | 0.05 | 0.06 |
| SJV^a^ South | 0.49 | 0.56 | 0.59 | 0.41 | 0.63 | 0.66 | 0.66 | 0.32 | 28\28 | 0.05 |
| Southern CA | 0.39 | 0.68 | 0.5 | 0.41 | 0.66 | 0.76 | 0.72 | 0.26 | 0.42 | 9\9 |

^a^Abbreviations are as follows: Half Moon Bay (HMB), Santa Barbara (SB), and San Joaquin Valley (SJV)

**Supporting Figures**

**Figure S1.** Inverse distance-weighted (IDW) interpolations of residual (from regression of genetic distance on Euclidian geographic distance) genetic distances (linearized *F*_ST_ or *D*_A_, respectively) associated with midpoints (for each pair of sites) for microsatellite (top 6 graphs) and mitochondrial (bottom 6 graphs) markers, based on actual data compared to 5 different random matrix permutations. Rows and columns were permuted identically in random order. Note that the actual case shows the greatest contiguous area covered by lower-than-expected genetic distances; only in the actual case do such areas correspond systematically to a landscape type, in this case, mountainous terrain.

**Figure S2.** Relationship between average (+/- SE) log probability of the data [LnP(X)] and the number of clusters (*K*) in 10 replicate runs of Structure at each *K* value (1–10) for 381 nonnative red foxes from California, based on the correlated allele frequencies model and 10,000 burn-in and 10,000 post-burn-in MCMC cycles.

**Figure S3.** Relationship between cumulative frequency (% of total) of genotypes corresponding to increasing numbers of cluster profiles as ranked from most to least frequent, illustrating that 11 profiles described assignments of 90% of all genotypes.

**Figure S4.** Hierarchy diagram of the 11 most frequent cluster profiles indicated by pathways connecting K2 and K8 clusters, illustrating lack of hierarchical structure for *K* < 6 due primarily to the cluster profile leading to cluster 1 at *K* = 8 associated with Morro Bay (light green).

**Figure S5.** Relationship between average (+/- SE) log probability of the data [LnP(X)] and the number of clusters (*K*) in 10 replicate runs of Structure at each *K* value (1–10) with a subset of 206 nonnative red foxes, including random subsamples of 20 foxes from each of the heavily sampled populations, East SF Bay, South and West SF Bay (combined), and Monterey Bay, based on the correlated allele frequencies model and 10,000 burn-in and 10,000 post-burn-in MCMC cycles.

**Figure S6.** Relationship between cumulative frequency (% of total) of genotypes corresponding to increasing numbers of cluster profiles as ranked from most to least frequent in a subset of 206 nonnative red foxes, including random subsamples of 20 foxes from each of the heavily sampled populations, East SF Bay, South and West SF Bay (combined), and Monterey Bay, illustrating that the top 6 profiles described assignments of 80% of all genotypes.

**Figure S7.** Hierarchy diagram of the 6 most frequent cluster profiles indicated by pathways connecting K2 and K8 clusters, based on analysis of 206 nonnative red foxes, including random subsamples of 20 foxes from each of the heavily sampled populations, East SF Bay, South and West SF Bay (combined), and Monterey Bay, from California according to admixture analysis with *K* = 8 genetic clusters in program Structure. The 6 highest-frequency cluster profiles indicate hierarchical structure, while the addition of 3 low-frequency clusters (dashed lines) was necessary to include all clusters at *K* = 6–8.

**Figure S8.** Bar graph indicating the ancestry fraction (q) apportioned to individual genotypes of 206 nonnative red foxes, including random subsamples of 20 foxes from each of the heavily sampled populations, East SF Bay, South and West SF Bay (combined), and Monterey Bay, from California according to admixture analysis with *K* = 8 genetic clusters in program Structure. Individual genotypes are arranged with “pure” assignments (q > 75%) to the left and admixed assignments to the right within each geographic sample group. Clusters indicated by light and dark shades of the same colors indicate those clustered together at *K* = 6 and 7 (gray/black) or *K* = 6 (blue).

**Figure S9.** Bar graphs indicating numbers of red foxes sampled from 7 sites assigned to *K* = 8 color-coded genetic clusters (i.e., Q_max_) in different sampling periods. Cluster colors correspond to Figures S3, S4. Time periods differ between sites to best reflect discrete breaks in temporal subsamples at each site.

**Figure S10.** Bar graphs indicating numbers of red foxes sampled from 7 sites carrying 1 of 9 nonnative mtDNA haplotypes in different sampling periods. Time periods differ between sites to best reflect discrete breaks in temporal subsamples at each site. The apparent changes in the San Joaquin Valley correspond to confounding with space, e.g., all the E-9s are from Bakersfield, whereas G-38 and N-7 were from the outlying areas of the countryside.
